# Combatting Intimate Partner Violence: Representations of Social and Healthcare Personnel Working with Gender-Based Violence Interventions

**DOI:** 10.3390/ijerph17155543

**Published:** 2020-07-31

**Authors:** Marcella Autiero, Fortuna Procentese, Stefania Carnevale, Caterina Arcidiacono, Immacolata Di Napoli

**Affiliations:** 1 OLV (Oltre La Violenza) Project, Naples Health Service, Piazza Nazionale, 95, 80143 Naples, Italy; marcella.autiero@gmail.com; 2 Department of Humanities, University of Naples “Federico II” Via Porta di Massa, 80133 Naples, Italy; fortuna.procentese@unina.it (F.P.); stefania.carnevale@unina.it (S.C.); immacolata.dinapoli@unina.it (I.D.N.)

**Keywords:** gender violence, professional representations, services contrasting gender-based violence, violence management, ecological approach

## Abstract

Intimate partner violence (IPV) has been declared a global epidemic by the World Health Organization. Although the attention paid to both the perpetrators and victims of gender-based violence has increased, scientific research is still lacking in regard to the representations of operators involved in interventions and management. Therefore, the following study explores how the representations of operators affect how gender violence can be managed and combatted through an ecological approach to this phenomenon, in addition to highlighting the roles of organizational-level services and their cultural and symbolic substrates. In total, 35 health and social professionals were interviewed and textual materials were analyzed by thematic analysis. The evidence suggests that services contrasting gender-based violence utilize different representations and management approaches. The authors hope that these differences can become a resource, rather than a limitation, when combatting gender-based violence through the construction of more integrated networks and a greater dialogue among different services, in order to make interventions designed to combat gender-based violence more effective.

## 1. Introduction

Gender violence against women and girls is a widespread international phenomenon, and the attention paid to gender violence interventions is sharply increasing.

The most pervasive and common manifestation of this phenomenon is intimate partner violence (IPV). Indeed, 1 in 3 women worldwide has experienced physical or sexual violence by an intimate partner or former partner [1,2].

The Istanbul Convention [3] provides specific and nondiscriminatory prevention and protection directives for women who are victims of many types of violence, encompassing “acts of physical, sexual, psychological, or economic violence that occur within the family or domestic unit, or between former or current spouses or partners, whether or not the perpetrator shares or has shared the same residence with the victim” [3] (p. 8).

In Italy, The National Institute of Statistics (Istat) [1], in a survey of 281 anti-violence centers, found that 43,467 women turned to them in 2017. Furthermore, 67.2% started a path to get away from violence and 63.7% had children (minors in 72.8% of cases). Foreign women made up 27% of those taken into care.

Although the Istanbul Convention [3] establishes that there must be an anti-violence center for every 10,000 inhabitants, the Istat analysis underlined that in Italy there was a center for every 20,000 inhabitants in 2017 (half of what it should be).

In Campania, which is the second worst Italian region by number of femicides, only 2374 women have turned to anti-violence centers.

Despite this, in the Neapolitan context, a health support network has also been activated–in the emergency rooms of the San Paolo and Loreto Nuovo hospitals in Naples, female victims of violence can also be reported on a psychological level after the violence suffered, allowing them to take advantage of healthcare support. The psychological report is sent to law enforcement agencies in order to support the victim from a legal point of view. The Cardarelli hospital in Naples provides a psychological support center for female victims of violence through the “Dafne” center. The Operational Unit of Clinical Psychology of Naples Health Services has set up a psychological support service for perpetrators of violence called “Oltre La Violenza” (OLV), whose primary objective is the taking charge of men who act physically, psychologically, economically, or sexually violent with current or former partners. OLV is the only public service within a healthcare context in all of southern Italy.

However, according to the literature, services oriented toward combatting IPV offer fragmented interventions that lack mutual integration and are affected by low levels of awareness regarding operators’ representations and prejudices [4,5,6]. There is a need to explore the context-dependent worlds of meaning in different intervention settings [7,8,9,10].

## 2. Literature Review

### 2.1. The Ecological Model from a Constructivist and Community Approach

In order to reduce the risk of careless simplifications concerning the phenomenon of gender-based violence, we utilize a situated approach in an ecological model [4,11,12] that studies the individual, relational, collective, and organizational domains, as proposed by the WHO [13].

This approach allowed us to detect the hidden roles of cultural and legal elements, as well as the organizational resources that feed or contrast IPV [14].

The quality of the network combatting gender violence influences the quality of both preventative and emergency interventions [15,16]. As a result, the construction of holistic and integrated guidelines may aid in overcoming the fragmentation of existing measures, highlighting the specificity of cases and contexts, as well as their intersections at multiple levels [4].

### 2.2. Research Concerning Health and Social Professionals Contrasting IPV

In Europe, while research concerning the victims and perpetrators of IPV is increasing, it is still very limited in regard to its representations of operators directly fighting violence against women and their perspectives [4,5,6,7,8,9,10,11,12,13,14,15,16,17]. An Italian study conducted in the Neapolitan area found that professionals working in general services represented a reference for citizens because they are part of a well-known public institutional network. However, it is important to highlight that general services do not have a specific mandate to combat gender-based violence, even though they often interact with both its perpetrators and victims. Conversely, operators of specific services dedicated to combatting IPV that belong to the nonprofit sector have often complained about a lack of institutional recognition for their services. Indeed, they expressed strong feelings of isolation that were similar to those of the violence victims with whom they work. These operators tried to overcome these obstacles through various initiatives based on the knowledge of and the personal collaboration among different services that are unable to reference standardized procedures [18].

### 2.3. IPV Social and Health Professionals’ Representations and Emotions

International and Italian studies have been carried out regarding general services, particularly concerning the roles of general practitioners (GPs) and public health operators who interact with victims of gender-based violence. A Danish study [19] showed that GPs lacked specific training and knowledge regarding DV, underestimating its prevalence and arguing that specific IPV training was unnecessary, too expensive, and of little relevance. Moreover, they perceived IPV as a personal problem that they should not address, unless explicitly requested by abused women [19].

In Italy, healthcare workers often demonstrate mechanisms of denial, minimization, and rationalization with respect to IPV [20]. In the Italian context, healthcare personnel often do not consider gender-based violence to be a matter of direct interest for hospital services, often due to a lack of time and space to devote to gender violence victims [21].

In this study, the obstetrics and gynecology staff who worked with gender violence victims reported strong concerns regarding the possible management of female victims of violence. Their primary concerns encompassed the absence of violence screening and protocols, resulting in situations where healthcare personnel were forced to rely solely upon their personal and individual listening skills when dealing with patients who were victims of gender violence. Moreover, Italian healthcare personnel often reported malaise and burnout in relation to their working conditions. Furthermore, they frequently expressed negative emotions, such as helplessness and anguish; as well as psychosomatic reactions, such as feelings of nausea, an empty stomach, sadness, anger, displeasure, resignation, and inadequacy, especially when acknowledging their patients’ violent experiences [22].

In this vein, when attempting to manage gender-based violence, healthcare workers sometimes experience emotional difficulties and chronic psychological defense mechanisms that prevent them from recognizing and assisting victims [20]. Procentese et al. [21] also noted that, in certain instances, they felt unable to offer help to victims, despite possessing relational and listening skills. In fact, a lack of policies, organizational protocols, and specific training affected healthcare workers’ beliefs, thoughts, and interventions regarding gender-based violence [21]. This finding highlights the need to include specific training regarding gender-based violence and its effects in the general education curriculum and to share the data from recent research and experiences in the services in order to create know-how in terms of taking charge of victims and perpetrators. Young Italian psychologists undergoing training frequently neglect the effects of IPV when examining the reasons behind children and mothers’ psychological discomfort. This ignorance enhances the invisibility of IPV, in addition to perpetrators’ attitudes that seek to justify their actions. However, it is important to highlight that prior studies have found specific training programs involving contrasting IPV to be very effective [23]. Therefore, it is essential to promote training courses in order to encourage a psychosocial management of IPV in all social and health services involved in the prevention and detection of IPV [24].

According to the literature, there is clear evidence regarding the negative impact of IPV on both the health of children and of mothers, although its effects are still largely ignored [25]. Moreover, children suffer as both witnesses of violence against their mothers and because they lack the care of their mother, who is unfortunately the victim of the daily violence. In addition to the organizational gaps and the poor training of health service personnel, the invisibility of IPV among operators is attributed to the common cultural power asymmetries [26,27,28] that characterize relationships between women and men, in which men wield greater levels of power [18,19,20,21,22,23,24,25,26,27,28,29,30].

Moreover, the literature emphasizes the need for specific training for operators who work with perpetrators of gender violence [5,31,32,33], particularly training opportunities to increase their reflexivity [34].The operators themselves support the need for the development of a dual perspective, oriented toward both oneself and others, in order to more effectively manage gender violence [33].

In particular, a specific professional background is essential in detecting seemingly invisible and unsuspected gender violence [35,36,37], as well as when assisting men in acknowledging the causes and effects of their actions and inviting them to find new strategies to express their negative emotions [5,6,7,8,9,10,11,12,13,14,15,16,17,18,19,20,21,22,23,24,25,26,27,28,29,30,31,32,33,34,35,36,37,38].

### 2.4. The Role of a Professional’s Gender in Managing IPV

The Istanbul Convention [3] established that victims of gender violence should be assisted by female workers when receiving related services. Furthermore, feminist literature emphasizes the importance of receiving assistance in a purely female context as being essential to providing more immediate intimacy, protection, support, and solidarity for female victims of gender violence [39].

Additionally, from a purely therapeutic rehabilitation perspective, Riccardi, Stanziano, and Nunziante Cesaro’s research [39] has also encouraged further reflection upon the dynamics that may be triggered among female victims of gender violence and the operators who assist them. From their perspective, there is a possibility that a sort of complicity could arise between victims’ needs for protection and operators’ desires to provide solutions, which could incentivize the passive adherence of victims to operators’ beliefs.

A recent study [33] highlighted how the operator’s gender can play a significant role in their experience working with abusive men. Female professionals [40,41] tend to be more empathetic to the plight of victims of gender violence, and the sociocultural impacts of gender violence against women may not prevent them identifying with, or even justifying, male perpetrators’ actions. Conversely, male operators, in comparison with female professionals, due to the self-reflexivity processes that they undergo as men develop an enhanced awareness of men’s roles in a patriarchal culture [33].

Therefore, this research will explore professionals’ personal and social representations to expand upon the existing knowledge concerning the interplay among their professional and cultural backgrounds and the different aims and structural organizations of the services that they provide. In order to pursue these goals, this study examines the current state of services designed to combat gender-based violence in a specific area, identifying both gaps and resources, in addition to listening to the professionals who work within the different service contexts.

## 3. The Research

### 3.1. Research Objectives

As previously highlighted, although the literature describes the representations, emotions, and management of male violence against women by operators working in general services, it has yet to explore these same elements in services specifically directed toward contrasting gender violence. Therefore, our research seeks to explore the representations, feelings, and management of operators providing three types of services in Italy, which are directed toward female victims of violence, perpetrators of gender violence, and families experiencing high levels of conflict, respectively. Our research aims to explore the similarities and differences among these three types services, which are further detailed below, in terms of their representations, feelings, and management of gender violence.

1. Anti-violence centers (CAV): Anti-violence centers provide support directed toward victims of gender violence. These centers form a part of “women for women” projects and are aimed at creating spaces to empower and support female victims of violence by offering psychological counseling, legal counseling, and professional guidance.
2. Centers for families (CpF): CpFs provide social and healthcare services to families experiencing elevated levels of conflict (Arcidiacono and Ferrari Bravo, 2009).
3. “Oltre La Violenza” (OLV): OLV is an Italian National Health Service program that targets the perpetrators of gender violence by encouraging meetings with and creating a therapeutic space for men who have subjected their current or former partners or wives to physical, psychological, economic, or sexual violence. “OLV should be understood as a possibility for operators and service users to rethink their emotional relationships, emphasizing respect for the otherness and dignity of both parties” (See: https://www.aslnapoli1centro.it/oltre-la-violenza).

### 3.2. Methods and Procedures

The participants consisted of 35 professionals (33 women and 2 men) between the ages of 27 and 66 years old, who possessed experience preventing, managing, and working with IPV perpetrators or victims. Participants included both volunteers and employed professionals with between 1 and 45 years of experience working to combat gender violence. They also occupied a variety of professional roles, encompassing psychologists, psychotherapists, social workers, ASL—Azienda Sanitaria Locale (the local public healthcare administrator) Naples 1 Center managers, family mediators, lawyers, and professional nurses. Participants were cultural, healthcare, and social personnel who work with victims and perpetrators of IPV regional healthcare and social services (Clinical Psychology Operational Unit ASL NA 1 District 31, Maternal and Child Operational Unit ASL NA 1, Minor and Family Services of the City of Naples, Center for Families ASL NA 1 Center), private psychology clinics, and anti-violence centers (CAV) organized by nonprofit associations and located at Cardarelli Hospital and in districts 24 and 26 of the City of Naples (Lilith and Le Kassandre).

Intentional purposive sampling was used to recruit study participants [42,43]. The researchers contacted participants by telephone to make appointments after identifying the most relevant contexts and accredited professionals in the Naples Metropolitan Area. Intentional purposive sampling is a nonprobabilistic procedure that selects a group of individuals for a sample in order to meet specific criteria. In the case of this study, the researchers utilized snowball sampling and only asked personnel who worked in services directed at preventing gender violence to participate in the study, recruiting them from a public list of government and nonprofit services offered in the Neapolitan area.

Participants’ interviews were carried out in a quiet and secluded environment, either at the researchers’ university or the interviewees’ workplaces. The interviews lasted between 30 min and 2 h each, with the average interview lasting 50 min. The research team was especially careful to respect the scheduling needs of interviewees when choosing the dates and times of interview meetings. The interviewees signed an informed and authorized consent form authorizing this study to use the data collected for research purposes.

According to focused interview [43] principles, the interviews were open [44,45], therefore both the researcher and the interviewee developed their themes of interest during the interview. These interview strategies provide a guided outline of the topics to be addressed, but not predetermined questions. This format facilitates opportunities for interaction between the interviewee and the interviewer. Conducting the study interviews required knowledge about the subject matter and the general and specific purposes of the research, as well as competence in conducting interviews.

This type of interview requires meeting the following criteria: (1) not to be a directive interviewer, by letting the flow of the interviewee’s thoughts run free as much as possible; (2) to ensure specificity (deepening the meanings that people attribute to phenomena considered in the research); (3) to be inclusive by considering all data that emerged, even if in contrast with what reported by the other interviewees; and (4) to adopt an in-depth approach by exploring the personal and inner aspects that characterize the interviewee’s experience. The research team considered this method useful by giving interviewees enough freedom to share their life stories, whilst being able to narrow down the narrative to specific area.

The interviewers were able to comprehend the innovativeness and specificity of the interviewees’ contributions, allowing them to add and expand upon novel research findings. They were required to demonstrate reflective competence, to suspend any judgments, and to maintain an active and stimulating attitude toward the interlocutor [34,35,36,37,38,39,40,41,42,43,44,45,46,47].

This research chose the following four thematic areas of interest for the interviews:

1. Representations of female victims of gender violence;
2. Representations of men who perpetrate gender violence;
3. Representations of the gender of operators who work with female victims of gender violence or male perpetrators of gender violence;
4. Representations of the purposes, problems, and strategies related to the services provided.

These thematic categories were identified in order to compare the current research with the most recent literature concerning the representation of operators working in anti-violence services. The interviews were audio-recorded and transcribed. Subsequently, this study utilized Braun and Clarke’s [48,49] thematic analysis method to analyze and interpret the interviews. Thematic analysis is a qualitative method that focuses on the qualitative aspects of the analyzed textual material [50].

In the context of this study, this implies “meaning and meaning-making, and viewing these as always context-bound, positioned, and situated; and qualitative data analysis [that] is about telling ‘stories’, about interpreting, and creating, not discovering and finding the ‘truth’ that is either ‘out there’ and findable from, or buried deep within, the data” [50] (p. 591).

Furthermore, this method assumes that texts and discourses do not have a single manifest meaning, but rather that all their explicit content can be linked to a specific context or situation. Utilizing thematic analysis allows researchers to analyze texts and to maintain awareness of any historical interactions or events that have played a crucial role in individuals’ psychological development, as well as in balancing group relationships. Consequently, studying themes allows for the description, analysis, sharing, and interpretation of the text in which they are contained. This technique consists of conducting a preliminary reading of the material, while maintaining a focus on the elements suggested by reference theories, in addition to those that emerged spontaneously during the research process.

In this regard, it is important to specify the research themes and to select vivid, significant, and synthetic extracts, which the researcher must reanalyze based upon the research question and findings from the literature before producing a final analytical report. However, it is important to emphasize that the primary aim of thematic analysis is to detect or infer meanings and not to measure the frequencies of the various categories that arise during the research process [48].

This study utilized thematic analysis because it allows for wide-ranging interpretations, as it is considered to be a type of “flexible” analysis, which permits researchers to play a decisive and active role in constructing the texts’ meaning. Furthermore, it should be viewed as a “descriptive method, because through a limited number of themes or categories, it describes what the data tells us” [51] (p. 182), and unlike other qualitative approaches it is essentially configured in a manner that is more similar to a method than a methodology.

In fact, according to Clarke, Braun, and Hayfield [52], a methodology represents a research approach built within a theoretical reference structure, “which informs the objectives and methods” (p. 224). In fact, Braun and Clarke [48] state that when a theory-driven approach is followed, as a result a (top-down) theoretical-deductive path is followed, in which the initial question does not change during the research [53].

The thematic analysis utilized during this study encompassed the following 5 phases:

5. Familiarization with the data;
6. Generating initial codes;
7. Searching for themes and subsequently reviewing them;
8. Defining and labeling themes;
9. Creating the research report.

The results of the thematic analysis of the study interviews are shown below, where they are also discussed in the context of the most significant and relevant literature.

## 4. Results

### 4.1. Representation of Female Victims of Violence

Concerning how different operators represent female victims of gender violence, anti-violence center operators tend to view female victims as being helpless and completely under their male partner’s control, often believing that removing them from their partners is the sole solution. Conversely, OLV and Center for Families operators typically describe female victims as “participants” in the violence dynamics, carefully noting that this depiction should not be confused with justifying or blaming women for gender violence. Examples of these divergent representations of female victims of violence derived from this study’s interviews are shown below.

“*From experience and from the various cases that we have had as an anti-violence center, women always have very low self-esteem [and] an identity I would not describe as destroyed, but as very, very worn, because they have constantly suffered episodes of violence for years*”.

(Psychologist, Anti-violence center (CAV), F, 34)

“*Certainly, the experiences we have had with victims of violence, in short, our own experiences, are based on this, aren’t they? We have opened a service for offenders because we already had experience with victims*”.

(Psychotherapist, Oltre la Violenza (OLV) project, F, 56)

In addition, some operators emphasized that female victims of gender violence can also exhibit collusive and manipulative behaviors:

“*It is a phenomenon that affects the couple, [and] it has to do with collusion that exists within the couple. Why not? One thing that is little talked about is that there are aspects related to women who can also be [involuntary] instigators of violence that they have to deal with and talk about. It should not be taboo to talk about this too. I think there is a need for skills, you cannot improvise. I do not speak of expertise, I speak of competence*”.

(Psychotherapist, OLV, F, 66)

### 4.2. Representations of Male Gender Violence Perpetrators

Among CAV operators, male perpetrators of gender violence are represented in an eminently polarized manner. These representations vary between viewing gender violence as a pure “*derivative of a patriarchal culture*”, in which violence is preordained—a purely cultural problem from Bourdieu’s perspective [26]—and understanding violence to be an individual “psychopathology.” These conflicting representations are illustrated by the interview excerpts included below.

“*Yes, but because it is the whole ‘village’ that speaks the same language. Why is it widespread? Because it is nourished by a culture that continues to possess power asymmetries. I ask myself about the best way to induce changes in men’s violent behaviors. It is difficult, among men, especially among adult men, because what do they have? They have the convenience of where they are, where their culture has placed them, and they do not feel discomfort. The dynamic behind this is precisely the act of joining a couple together in the context of power asymmetries that are ‘preordained’ by external forces. In other words, gender violence is already spelled out. And it is a short circuit among couples, because having a society organized based on these power asymmetries makes it clear that I enter a couple with a cultural background that values me (a woman) less than a man, who is considered to be superior to me*”.

(Sociologist, CAV, F, 60)

“*In my opinion, the primary characteristics of violent men are their obsessive personality traits (also, their inability to manage emotions, but these are connected), because when these dynamics occur, men must be considered pathological, because they are pathological*”.

(Psychologist, CAV, F, 30)

In many cases, the desire to not want to deal with perpetrators of gender violence emerges, although some professionals are bound by current legislation, which prohibits those who work with female victims from also working with male perpetrators. However, others express a categorical refusal to work with perpetrators of gender violence, often encapsulating their viewpoints through statements such as the following interview excerpts.

“*Fortunately, it does not concern me.*”

“*Eh, I have never asked myself about this problem because I look at the other side of it. I have to be honest, at this moment it is difficult to provide an answer in this sense*”.

(Lawyer, CAV, F, 43)

“Female-centered” operators often show little interest in the suffering that perpetrators of gender violence might experience. However, despite this “predisposition”, the need to deal with perpetrators is considered, as illustrated below by one interviewee’s characterization of this context.

“*It is obviously understood that we take sides, but going beyond this, it is stupid not to want to understand what is going on altogether*”.

(Psychotherapist, CAV, F, 37)

Indeed, by working in direct contact with men, OLV and CpF operators report a more defined representation of the perpetrators of gender violence. This allows them to better understand gender violence perpetrators’ emotional difficulties, which are primarily related to complications in managing anger, fear, and anguish, as well as communication problems and elevated levels of psychological distress, as illustrated by the following interview excerpts.

“*Poor management of frustration linked to this, an absence of development of one’s autonomy and individualization, an inability to be in relationship with others, and poor anger management, I believe these are also linked to one’s social context*”.

(Psychologist, Center for Families (CpF), F, 57)

“*The men I work with show communication failures. I mean, they do not really try to talk [with their partners], they give them a smack. They hit them*”.

(Psychotherapist, OLV, F, 62)

Regarding the reasons why men would turn to gender violence prevention services, many CAV operators show strong reticence concerning men’s desire to change, as depicted below.

“*Well, I have some doubts, because culturally men do not recognize violence. They do not recognize themselves as perpetrators of violence. And they cannot even understand the violent devices that are contained within their actions, which range from the slightest... verbal criticisms of their partners, to the most severe actions, such as physical aggression or femicide*”.

(Sociologist, CAV, F, 60)

OLV and CpF operators more frequently emphasize the need for perpetrators of gender violence to turn to services to care for their psychological health than CAV operators, underscoring their fears of losing loved ones. This finding is illustrated by the affirmations made by the interviewed operators, shown below.

“*Let’s say, in my opinion, they can still possess the motivation to get better because these people are not well, in the sense that they do not have a satisfactory healthy relationship. However, in a certain way, they are simply held back by their partner, such as negative people, who do something negative, so in my opinion, there is always suffering*”.

(Psychotherapist, OLV, M, 31)

“*Some men are aware that these dynamics lead to estrangement, to the loss of their partners, and sometimes even of their children. This awareness could possibly lead them to acknowledge what is being done and induce them to do something to change their relationships and interactions with their partners*”.

(Psychotherapist, OLV, F, 31)

### 4.3. Representations of the Operator’s Gender When Assisting Female Victims of Violence and Interacting with Perpetrators of Gender Violence

All interviewees agreed that female victims of gender violence who seek assistance must interact with a female operator during the intake process, as stipulated by the Istanbul Convention (2011). Working with female operators could allow women to more easily identify with gender violence intervention operators, while initially working with a male operator could evoke trauma among female victims of gender violence.

“*Finding female operators is fundamental for female users. Coming to a center where all operators are women, in short, allows female victims of gender violence to identify with the operators, as well as to understand the difficulties that are inherent to our cultural and political context*”.

(Psychologist, CAV, F, 38)

“*In this sense, I think that, for a woman, [initially] meeting with a female operator is important, because meeting with a male operator could cause them to relive past trauma. We are talking about women that still show signs of post-traumatic functioning, and therefore, in some way, a female reference could certainly be positive for them, in order to avoid making them relive feelings of trauma, or perhaps of anguish*”.

(Psychotherapist, CAV, F, 39)

Following the initial phase during which female victims must be welcomed by another woman, some operators, particularly OLV and CpF operators, claim that female victims can also work with male operators at a later stage, as illustrated by the interviewee’s affirmation below.

“*We are not talking about a woman arriving at the emergency room, right? There, it is different, and it is clear that there is a need for female operators to welcome [victims of gender violence]. However, we are talking about professional listening, so for now I do not see the [inherent] need for either a male or female operator*”.

(Psychotherapist, OLV, F, 61)

Regarding the gender of operators working with female victims of violence, some CpF operators propose a co-management strategy by a pair comprised of a male and a female operator, which could offer the opportunity to confront different models and relationship styles.

“*Working in pairs would be better, at least at the Center for Families where we work. The therapeutic ‘couple’ somehow reproduces the parenting one, and that gives others the opportunity to visualize a relationship model, because we are obviously talking about a relationship*”.

(Social worker, Minor and Family Services, F, 44)

Concerning working with perpetrators of IPV the majority of operators say that their professionalism and specific on-the-job training are more important than their gender. Conversely, certain studies have indicated that the operator’s gender can influence their therapeutic relationship with gender violence perpetrators, underscoring a need for positionality and reflective concern [47].

### 4.4. Representations of Intervention Strategies among Different Services

In regard to perpetrator-focused intervention strategies, all operators indicated that they were in favor of such strategies, but the operators’ perspectives differed among those providing different services.

Concerning the representations of CAV operators, there is a sort of “reserved agreement”, primarily due to economic motives. This reflects their feelings of resignation in the face of the difficulties involved in changing gender violence perpetrators’ behaviors, while also accentuating conflicts regarding the already limited funding dedicated to anti-violence centers for women. Therefore, from the perspective of CAV operators, funding directed toward assisting men who perpetrate gender violence is often seen as a “wasted” or “useless” investment rather than a resource, particularly in a context in which the need to provide for their victims figures as a more pressing concern.

Moreover, CAV operators confirmed their belief in the importance of working with gender violence perpetrators, as these interventions are aimed at communicating with the “actors of a violent culture”—the so-called “executioners”. However, they viewed perpetrator-focused interventions as being primarily important in reducing the number of victims (including both women and children), while continuing to express “skepticism about the success of this intervention strategy”, as illustrated below.

“*Eh, but in short, I do not think it is useful, because there is already a lot of impunity for these men, never mind!*”.

(Psychotherapist, CAV, F, 39)

OLV and CpF operators were more likely than CAV operators to voice a sense of “responsibility” to work with gender violence perpetrators. They frequently sought to comprehend gender violence beyond the mere “victim–perpetrator” dynamic, in order to understand the suffering that violence causes to perpetrators themselves, as well as among all family members, despite the fact that this is frequently very difficult to accept, as expressed by many interviewees.

“*Violence perpetrators are part of a relationship, which can lead to violence, although it may encompass past, lived experiences and an entire personal history that may be imbued with violence. Experiencing and having experienced these types of situations surely causes suffering among many perpetrators as well. Therefore, I do not necessarily see them from the victim–victimizer perspective, but rather as people who have constantly lived in violent contexts, resulting in these men becoming violence carriers. Therefore, I have no problem working with violence perpetrators, who are surely the most difficult to get in contact with*”.

(Psychotherapist, OLV, M, 31)

Among OLV operators in particular, perpetrator-focused interventions are a “health-related mission” that must take advantage of perpetrators’ healthy characteristics and their resources.

“*But we are always thinking about gender-based violence as a phenomenon that must be countered, because it is not a good thing, is it? Because you do not have to beat your wife, because you do not have to beat your children, because you have to be a good father, a good husband, and you have to stop provoking. We still behave as if there was no need for professional intervention, and we act as if it were a behavior that is surely changeable, so it is as if we were always quick to judge. I mean, that is not good. Now, you are a man or a woman, you are a male psychologist, or you are a female psychologist, you are a male social worker, or you are a female social worker—it is always the same thing*”.

(Psychotherapist, OLV, F, 61)

## 5. Discussion

The operators’ representations are very similar, but at the same time their differing backgrounds, social mandates, and the types of service users with whom they interact highlight specific peculiarities among different operators. First, substantial differences are evident among general services operators, who lack specific training and ad hoc perspectives to detect and work with IPV victims and perpetrators. Most of the interviewees pay special attention to the manipulation and triangulation that operators dealing with IPV face [54,55]. They could be induced to side with either the male perpetrators, female victims, or both, therefore reproducing the same violent relationship dynamics when providing gender-violence-related services.

In general, given the nature of their services, CAV operators’ representations express an emergentist approach focused on the need to protect and urgently provide for women and their children, which is centered on intake services; psychological, legal, and professional guidance; and self-help groups. Conversely, OLV operators’ representations outlined a dual approach focused on both preventive and long-term restorative services. Preventive services utilize shelter listening services that are open to requests from men who seek to stop their potentially abusive behavior before they become violent. The long-term restorative services offered by OLV operators are directed toward men who have already acted violently, focusing on relationship contexts where full-blown acts of violence have already taken place. In contrast with CAV and OLV operators, CpF operators’ representations take a more preventative approach that focuses on the couple and attempts to rectify profound familial differences, which in some cases can lead to violence. In this regard, CpF operators’ work with couples could be seen as preventing the escalation of violence and as a preliminary step in gender-based violence interventions.

In addition to the aforementioned general characteristics regarding CAV, OLV, and CpF services, it is important to highlight the following differences among them. Regarding the representations of female victims of violence, CAV operators often described them through an individualistic lens, depicting women who passively suffer from gender violence. However, in contrast, OLV and CpF operators frequently viewed gender violence through a relationship-focused lens, highlighting couples’ reciprocal bonds. Despite these differences among operators, it is fundamental to underline that all interviewees stressed the importance of separating the respective psychological and judicial consequences of gender violence in order to avoid the risk of confusing the use of a relationship-focused lens with affirming the co-responsibility of the victim. This is due to the fact that 31 interviewees were psychologists (62%), and from their point of view it was important to understand the couple’s dynamics. However, from a legal point of view, there can only be one victim and one perpetrator.

Concerning managing female victims of gender violence, CAV operators faced the task of protecting them and removing them from a dangerous situation. Their work was primarily aimed at protecting women’s welfare, from both a psychological and legal viewpoint, in addition to restoring the victims’ autonomy and helping them escape violent relationships.

However, CAV operators claimed that many women who turn to them abandon the therapeutic process because they decide to return to their partners or to withdraw their legal complaints. Some of them report that the partner has changed, while others report having financial difficulties. An interdependence is created in the couple, which is both highly psychological and economic.

Moreover, a further goal is to maintain the family unity, and even if oppressed by family burden they are worried about the risk of damaging their children’s wellbeing with the effects of the family disruption, Therefore, this dropout risk clearly exists, and a merely “emergentist” approach does not allow it to be managed effectively.

However, there is no doubt among interviewees regarding the essential need for emergency interventions for IPV victims, as its brutality requires rapid and careful handling in a purely female context, which can prove to be a fundamental resource in terms of facilitating profound intimacy, self-protection, support, and consolidation [39,40,41,42,43,44,45,46,47,48,49,50,51,52,53,54,55,56]. In this regard, all operators believe that it is essential that only women provide intake services to gender violence victims in order to avoid causing further trauma to victims at the hands of the services themselves.

Nevertheless, following the initial intake phase, working exclusively with women may constitute a limitation in assisting gender violence victims. As stated in a prior clinical study by Riccardi et al. [39], accepting rigid models risks a lack of flexibility to act consciously, above all in threatening and dangerous emergency contexts characterized by a need for short, rapid response times. Furthermore, in the context of emergency, gender-based violence interventions may stifle female victims’ needs for independence and fear of freedom, conversely supporting their passive adherence to the operator’s vision of women’s familial and societal roles.

In accordance with these findings, Riccardi et al. [39] emphasized the importance that male operators play for victims of gender violence during the subsequent phases following victim intake. Male operators may play a “reclaiming” role that is aided by a setting more oriented toward neutral listening rather than mutual identification. This dynamic aids in the development of more profound emotional dimensions, characterized by positive transfers that could play an important role in assisting female victims of violence in achieving autonomy and self-determination. Moreover, developing a clinical relationship with male professionals could change female victims’ experiences in relation to men, in a context where their relationships with their male partners were characterized by threats, aggression, and persecution [39]. This previous point may help explain the dropout rates experienced by anti-violence centers, illustrating how they could be countered in later phases of gender violence services through articulated and differentiated interventions.

Regarding the management of female victims of violence, OLV and CpF operators were more likely than CAV operators to support the argument that following the victim intake phase during which a female operator is necessary, either male operators or a pair of male and female operators are capable of adequately assisting female victims of gender violence. In this regard, Riccardi et al. [39] hypothesized that male operators could play an important “reclaiming” role that could help female victims in cultivating differentiation and autonomy. Through a setting oriented by “neutral listening”, male operators could simultaneously allow female victims of violence to develop positive experiences in relation to men, which had previously been dominated by aggression and persecution. In this regard, the data findings must be interpreted within a critical framework [57] that attaches importance to both operators’ positions and intersectionality. In fact, when managing a phenomenon so steeped in cultural dimensions, it is necessary to consider the value dimensions underlying the strategies for understanding that phenomenon and any relevant interventions [47].

A long-term vision, and above all the development of prospective intervention strategies, could permit for long-lasting and long-term gender violence management that allows women to explore their emotionally collaborative behaviors and their ability to manage them, while also attempting to reduce gender violence intervention services dropouts. From this perspective, the operator’s gender would no longer make a difference, as both male and female operators undergo specific professional listening training.

Indeed, regarding representations of men who commit gender violence, CAV operators possess a more fragmented and less unitary viewpoint, as they tend to understand gender violence through a polarizing lens focused on two extremes of a continuum. On one end, they view gender violence as a purely cultural phenomenon, resulting from a patriarchal culture that justifies and legitimizes IPV and which necessitates the re-education of men who perpetrate gender violence. At the other extreme, CAV operators view the phenomenon of gender violence as being more individualized and violence perpetrators as possessing psychopathological disorders and as needing treatment.

Consequently, the resulting understanding of gender violence is characterized by the necessity to remove, punish, and treat perpetrators of gender violence, while saving and protecting female victims of violence. However, it is very likely that this representation, in addition to adhering to specific mandates and training regarding female victims, is influenced by the types of service users with whom CAV operators work with daily. Many of the female victims who arrive at CAVs are in an emotionally fragile state as a result of having been subjected to violence for years. As a result, the counter-transferal experience lived by CAV operators also plays a fundamental role, as “taking care of” or “worrying about” perpetrators of gender violence could signify “betraying” the women who turn to them for assistance. Such actions could elicit strong feelings of guilt among CAV operators, as confirmed by the literature [18].

On the other hand, the specific knowledge that OLV and CpF operators possess about both violence perpetrators and female victims leads them to have a more relationship-centric view of gender violence, often focused on triggers resulting from specific couple dynamics. This approach could be explained by the “double interaction” that characterizes these operators’ work, as they work closely with both members of the relationship. In the case of OLV operators, they also work with gender violence victims in other programs, while CpF operators work closely with couples experiencing conflict.

Regarding the management of violence perpetrators, both the study interviewees and earlier research [33,34,35,36,37,38,39,40,41,58] found no differences in operators’ representations based upon their gender. This study considers and discusses factors related to how the gender identity of gender violence intervention personnel could influence their professional interactions with both victims and perpetrators. However, CAV operators often were resistant to working with gender violence perpetrators for both economic motives and due to a skepticism that male perpetrators were truly capable of change. Regarding their economic reasons, many CAV operators believe that funds spent on men could deprive female victims of violence of already scarce resources.

However, despite the reluctance of CAV operators, perpetrator-focused gender violence interventions are necessary to neutralize perpetrators and consequently reduce the number of gender violence victims. In this regard, both OLV and CpF operators view working with gender violence perpetrators as a way to not only reduce the number of victims, but also to assist perpetrators in overcoming their own suffering. This strategy could allow gender violence perpetrators to rediscover and utilize their internal resources and the healthy aspects of themselves to reduce their own suffering, as well as acts of gender violence.

Finally, in the operators’ words, the divergent nature of gender violence intervention services enhances the fragility of their users’ familial relationships by focusing on individual actors (women, men, or children) from an “emergentist” perspective. This approach is often fueled by a lack of economic resources and risks crystallizing people in “rigid roles” (violence perpetrators to be dismissed versus victims to be rescued), which do not allow for the development of an effective and holistic dialogue among the various gender violence intervention services.

## 6. Conclusions

Our research focused on the institutional mandate and the interactions among gender-violence-related services within the framework of the Italian legislative and political system, as viewed from an ecological and constructivist approach to IPV [4]. As a result, this research seeks to “challenge” existing operational models, exploring the real-life conditions of specific services, while identifying the shortfalls, resources, and worlds of meaning (representations) that inform and are informed by intervention settings [7,8,9,10].

The study results show that the more emergentist approach of CAV operators and the long-term preventive approaches of OLV and CpF operators are likely influenced by the respective social mandates of each of the services, but that they also influence violence management strategies themselves. The risks involved in the utilization of different approaches lie in the operators’ differing representations and language, which sometimes contrast and contradict with one another. Therefore, these dimensions could partially explain the fragmentation of interventions aimed at contrasting IPV.

In the case of CAV operators, violence management focuses on protecting women and their children from serious and immediate danger, “setting aside” perpetrators and any possible assistance that could be provided to them. However, in the case of OLV operators, working with gender violence perpetrators is a priority that aims to “also care for” the “suffering” that these men have been subjected to, in order reduce their violent behavior. Finally, CpF operators’ work with couples is aimed at preventing violence within families, in an attempt to ensure that high levels of conflict among couples will not result in IPV. As a result, all three services approach the problem of gender violence through different users, at different times, and using different methods.

On the one hand, these divergent approaches can be seen as resources, because they consider the extreme complexity and heterogeneity of the phenomenon of gender violence. On the other hand, this divergence can explain the fragmentation of violence representations and contrasting policies. Therefore, we hypothesize that the limited integration of gender violence intervention services can also be understood as a fragmentation of the operators’ representations, as well as a lack of strong networks and standardized specific protocols, as outlined by Procentese et al. [21]. An effective network of integrated services that communicate with one another could help activate a process of violence “disclosure” and prevent its reoccurrence. While Arcidiacono and Palomba’s [59] research concerned child abuse, their findings may also be applicable in enhancing the effectiveness and efficacy of IPV intervention services.

However, this study was carried out among operators who act in specific, situated realities, which limits the generalization of its findings. As a result, the study analysis possesses a contextual nature, and it would be interesting to expand its scope at the national and international levels regarding the different types of services offered to combat gender-based violence. Furthermore, despite the need for diversified interventions in practice, greater importance should be given to more uniform and homogeneous representations among gender violence intervention operators at a network level. Moreover, future research should investigate whether operators need to be more cohesive in an international context and on an organizational level, as well as if they are capable of finding common solutions to understand the complexities of IPV and to contrast the divergence in policies and practices for managing gender-based violence.
